# Transition from Surface to Hopping Conduction in Stacked Nonepitaxial Bi_2_Se_3_ Dual Thin Films

**DOI:** 10.1021/acsomega.5c10654

**Published:** 2026-03-11

**Authors:** Kuan-Han Wu, Cheng-Yi Cheng, Bo-Chien Liao, Jian-Jang Huang

**Affiliations:** † Graduate Institute of Photonics and Optoelectronics, 33561 National Taiwan University, Taipei 10617, Taiwan; ‡ Department of Electrical Engineering, 33561 National Taiwan University, No. 1, Roosevelt Road, Sec. 4, Taipei 106, Taiwan

## Abstract

We investigate quantum transport in topological insulators through nonepitaxial thin film stacking. A KOH­(potassium hydroxide)-assisted mechanical transfer is developed for fabricating stacked nonepitaxial Bi_2_Se_3_ dual thin films. While deliberate rotational misalignment is introduced during stacking, X-ray diffraction confirms the preservation of crystallinity and strong *c*-axis orientation. Single thin-film samples exhibit a metallic-to-activated transition near 130 K, indicative of surface-dominated transport. In contrast, the dual thin-film stack shows a nonmonotonic resistance minimum at 50 K, followed by an upturn well described by a three-dimensional variable-range hopping model (*R* ^2^ ≈ 0.98), suggesting disorder-driven localization. Magnetotransport measurements further reveal suppressed linear magnetoresistance and enhanced weak antilocalization, indicating disrupted surface coherence due to interfacial hybridization. These results demonstrate that stacking-induced disorder fundamentally alters the transport regime, favoring hopping conduction over coherent surface states. This work provides a platform for engineering quantum transport through nonepitaxial stacking in topological systems.

## Graphical abstract

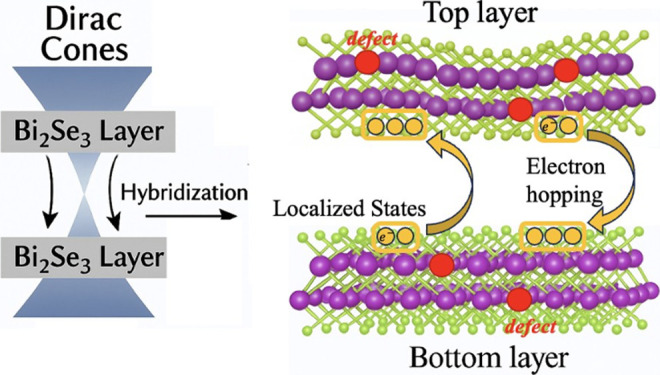

## 1 Introduction

Topological insulators (TIs) constitute a class of quantum materials^1^ distinguished by their insulating bulk and robust metallic surface states,^2,3^ which originate from nontrivial topological order^4^ and are protected by time-reversal symmetry.^5–7^ Among the available TIs, Bi_2_Se_3_ is a model compound with a relatively simple band structure, characterized by a single Dirac cone^2,8,9^ on the surface, and a sizable bulk bandgap (∼0.3 eV).^2,10^ The features allow Bi_2_Se_3_ to retain topological surface conduction over a wide temperature range, making it an ideal TI material for studying fundamental aspects of topological transport in solid-state systems.

In two-dimensional (2D) materials, reduced dimensionality and strong quantum confinement critically influence charge transport behavior. The high surface-to-volume ratio increases the impact of interface quality, disorder, and dielectric environment on conduction. For functional applications, such as field-effect transistors, low-noise sensors, and tunneling devices,^11,12^ precise control over temperature-dependent transport characteristics is essential. Compared to conventional 2D semiconductors like MoS_2_ ^13^ or black phosphorus, TIs exhibit a qualitatively different transport mechanism that stems from the coexistence of gapless surface states and a gapped bulk, resulting in unique resistance signatures and crossover phenomena that are not observed in typical semiconducting 2D systems^14–16^

Furthermore, 2D materials’ transport behavior strongly depends on layer number and material type,^17^ particularly in temperature-dependent resistance (R–T) measurements. For instance, monolayer graphene demonstrates nearly temperature-independent resistance at high carrier densities due to its metallic Dirac dispersion, but shows weak localization at low temperatures when disorder is present.^18^ In contrast, few-layer black phosphorus behaves as a narrow-gap semiconductor with highly anisotropic transport, where increasing thickness reduces the bandgap and leads to thermally activated transport transitioning to hopping-like conduction at cryogenic temperatures.^19^ Transition metal dichalcogenides (TMDs), such as MoS_2_ and WS_2_, typically exhibit semiconducting behavior with a significant increase in resistance at low temperatures due to Schottky barriers and trap-assisted tunneling.^20^

These behaviors contrast sharply with those observed in TIs like Bi_2_Se_3_, where both gapless surface states and a gapped bulk coexist. In multilayer TI systems, stacking may induce interlayer hybridization and topological gap opening, resulting in a fundamentally different transport mechanism from conventional 2D semiconductors. However, the impact of nonepitaxial stacking on such transport properties remains insufficiently explored, particularly concerning disorder-induced localization and dimensional crossover in resistance behavior.

One major challenge in studying multilayer TIs is the controlled fabrication of clean and well-defined stacked structures. Molecular beam epitaxy (MBE) offers high crystallinity and atomic precision, but its scalability is limited. In contrast, chemical vapor deposition (CVD) enables large-area growth but typically lacks precise layer control.^21^ Mechanical transfer techniques, especially those involving van der Waals materials, have been widely used to assemble multilayer heterostructures,^22^ including Bi_2_Se_3_, using methods such as polymer support delamination or wet chemical lift-off. However, the influence of transfer-induced disorder, rotational misalignment,^23^ and interface quality on quantum transport has not been systematically addressed, particularly for nonepitaxially stacked^24^ TI thin films.

This work presents a method to fabricate stacked nonepitaxial Bi_2_Se_3_ thin films by combining CVD growth with a KOH (Potassium Hydroxide)-assisted mechanical transfer process. This approach enables vertical stacking with rotational misalignment while preserving crystallinity, providing a clean platform to explore stacking-induced transport phenomena. Structural and electrical measurements reveal that interfilm disorder^25,26^ and hybridization^20^ suppress coherent surface transport and the emergence of three-dimensional variable-range hopping (3D VRH) conduction^27–29^ at low temperatures. Our results demonstrate that nonepitaxial stacking thin films fundamentally modify the quantum transport in TIs, offering insights into interfacial localization and transport dimensionality in topological multilayer systems.

## 2 Method

To realize such stacked Bi_2_Se_3_ dual thin films, we employed a two-step chemical vapor deposition (CVD) process to synthesize high-quality thin films on Al_2_O_3_ substrates. Often combined with pressure control and postannealing, CVD techniques have been widely adopted for fabricating large-area, low-defect Bi_2_Se_3_ films tailored for quantum transport applications.^30^ In this work, Bi_2_Se_3_ thin films were synthesized using a two-step CVD process in a horizontal quartz tube furnace, as schematically illustrated in Figure 1a. High-purity Bi_2_Se_3_ powder (99.999%, 600 mg) was placed at the center of the furnace as the source material. At the same time, Al_2_O_3_ (0001) substrates were symmetrically positioned at both ends of the quartz tube, approximately 30 cm from the center, to for thin film deposition.

**Figure 1:**
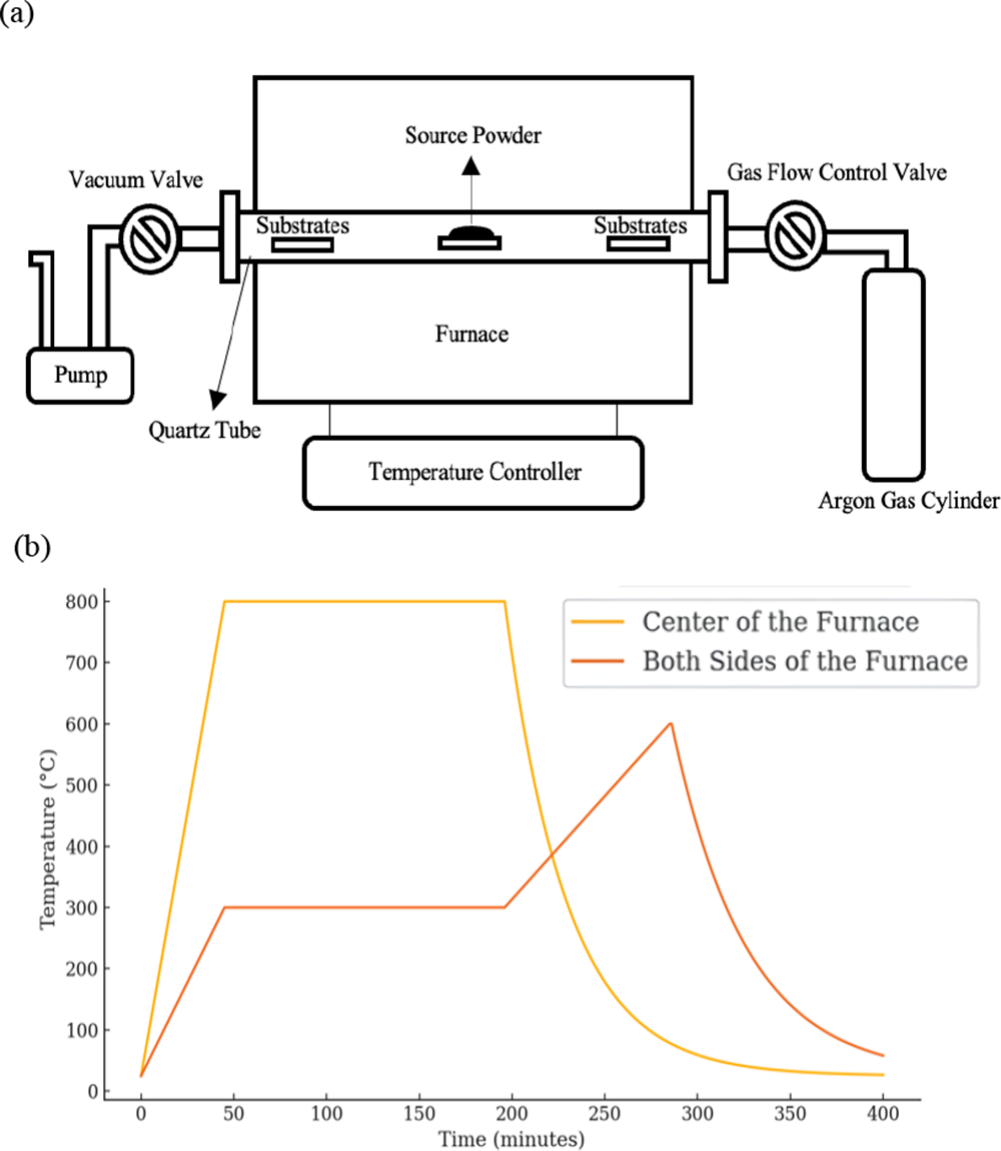
(a) Illustration of the horizontal quartz-tube CVD setup used for Bi_2_Se_3_ thin-film growth. High-purity Bi_2_Se_3_ powder (99.999%, 600 mg) was placed at the center of the furnace as the source material. At the same time, Al_2_O_3_ (0001) substrates were symmetrically positioned approximately 30 cm from the center at both ends of the tube to collect the deposited films. (b) Temperature ramping profiles along the furnace tube. The central heating zone reached 800 °C, enabling Bi_2_Se_3_ sublimation, whereas the substrate zones stabilized at ∼300 °C for film deposition. We then performed the postdeposition annealing step by reversing the temperature gradient to desorb residual surface impurities and enhance crystallinity.

Initially, we vacuumed the system to a base pressure of approximately 5 × 10^–2^ Torr. Argon (Ar) gas was then introduced at a flow rate of 150 sccm to raise the chamber pressure to about 250 Torr. We further reduced the pressure below 5 × 10^–3^ Torr to remove residual gases and surface contaminants. As shown in Figure 1b, the furnace temperature was adjusted so the central zone reached 800 °C to sublimate Bi_2_Se_3_. In comparison, the substrate regions stabilized at approximately 300 °C, providing an optimized environment for thin-film deposition. The growth duration was 150 min to ensure uniform coverage. Following deposition, a postgrowth desorption step was carried out by allowing the center zone to cool while simultaneously heating the substrate zones to ∼650 °C. This inverted temperature gradient enabled the desorption of residual surface species, yielding smoother Bi_2_Se_3_ films with reduced surface defects, which is favorable for subsequent thin-film stacking and electronic transport studies.^30^

The CVD-grown Bi_2_Se_3_ thin films were mechanically transferred via a potassium hydroxide (KOH)-assisted process to achieve the stacked thin-film structure. A poly­(methyl methacrylate) (PMMA) layer was first deposited on the film surface by spin coating for mechanical support. After drying, the PMMA-coated samples were immersed in diluted aqueous KOH solution to initiate lateral underetching.^31,32^ During this process, the etchant gradually diffuses beneath the film–substrate interface, weakening adhesion and allowing mechanical lift-off. This PMMA-assisted KOH delamination method has been widely adopted for transferring two-dimensional materials, including Bi_2_Se_3_ and MoS_2_, due to its compatibility with large-area films and ability to preserve crystallinity^33–35^

Three KOH concentrations (13.05, 17.80, and 21.75 wt %) were tested to optimize the delamination conditions. We used optical microscopy to monitor the transfer behavior. Lower concentrations yield larger delaminated areas, but more surface wrinkles and clusters, likely due to stress relaxation or residue formation. Among the concentrations, 21.75 wt % achieved a balance between delamination efficiency and surface uniformity, and was selected for dual thin-film assembly (see Figure S1).

After delamination, we floated the PMMA-supported Bi_2_Se_3_ film on the water surface. Dual thin-film stacking was achieved by gently retrieving the floating film with another CVD-grown Bi_2_Se_3_ film on an Al_2_O_3_ substrate. The stacked sample was dried under ambient conditions and baked at 90 °C for 30 min to ensure smooth interfacial contact without deformation. Finally, the PMMA support was removed using acetone to minimize residual contamination.

Standard photolithography defined electrode structures on single and dual thin-film Bi_2_Se_3_ devices. An electron-beam evaporation deposited a metal stack of Ti (10 nm)/Au (200 nm), with Ti as an adhesion layer. The metal lift-off process yielded clean and well-defined contact patterns without visible residues. A four-probe electrode configuration was patterned at the film corners for the single thin-film devices to facilitate standard electrical characterizations such as resistance and Hall measurements. In the dual-film devices, electrodes were aligned precisely over the overlapping region of the top-transferred Bi_2_Se_3_ film, enabling localized probing of vertical transport and interfilm coupling. The details of the electrode design are provided in the Supporting Information (Figure S2).

## 3 Results and Discussion

Before electrical measurements, we examined the crystallinity and phase integrity of the Bi_2_Se_3_ thin films to ensure that the fabrication and transfer processes did not degrade the structural properties. X-ray diffraction (XRD) measurements were thus performed on three representative configurations: an as-grown film, a transferred film, and a dual-film stack. Figure 2a presents the XRD patterns of the as-grown thin film. Distinct (00l) reflections, including (003), (006), (009), and higher-order peaks, are shown. They are associated with the rhombohedral *R*3̅*m* phase.^36,37^ The sharp and intense (006) peak at 2θ ≈ 19.0° indicates strong *c*-axis orientation and excellent out-of-plane crystallinity.^38^

**Figure 2:**
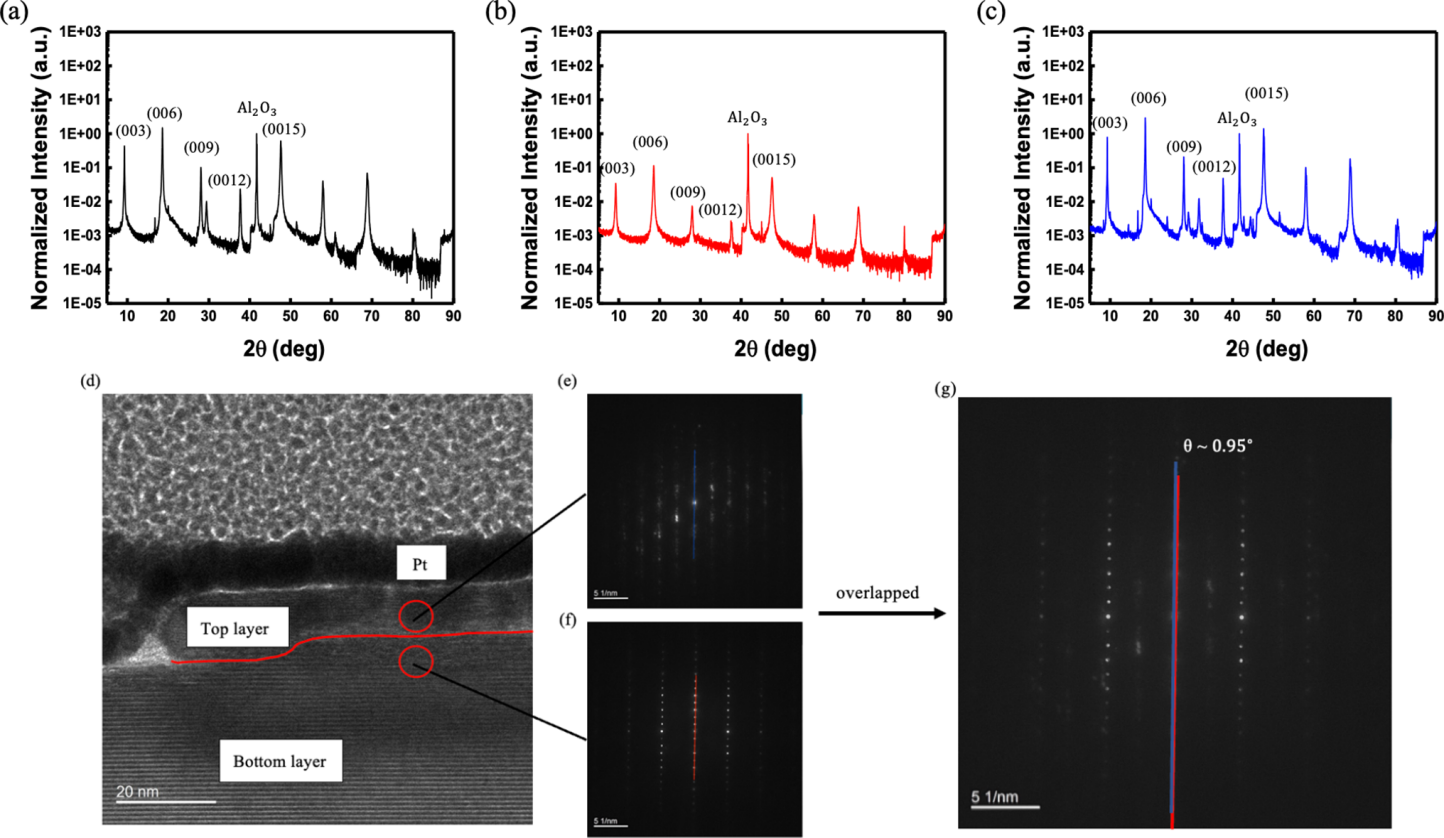
X-ray diffraction (XRD) patterns of Bi_2_Se_3_ thin films in three configurations: (a) as-grown thin film (black), (b) transferred thin film­(red), and (c) stacked dual thin films (blue). All samples exhibit (00l) reflections consistent with the rhombohedral R–3m phase. The transferred film shows reduced intensity without phase degradation. The dual-film sample displays enhanced peak intensities at (006) and (0015), indicating preserved crystallinity after stacking. (d) Cross-sectional TEM image of the stacked thin film. The two Bi_2_Se_3_ thin films are clearly distinguishable: the top film (above the red line) and the bottom one (below the red line). The red circles mark the approximate locations where we acquired the NBD patterns in (e,f), corresponding to the top and bottom films. (g) The overlapped diffraction spots are aligned vertically, indicating a small stacking angle of ∼0.95° between the two films.

The transferred thin film shown in Figure 2b, obtained by delaminating an as-grown film using a KOH-assisted lift-off process and redepositing it onto a fresh Al_2_O_3_ substrate, retains the same (00l) peak sequence. However, the overall diffraction intensities are significantly reduced, likely due to diminished surface uniformity, microwrinkles, or partial film coverage introduced during mechanical transfer. No additional peaks are observed, confirming that the process preserves the phase composition without introducing crystalline degradation.^39,40^

The dual thin-film sample (blue), created by stacking a transferred film atop an as-grown one, also preserves the rhombohedral phase. Figure 2c shows the enhanced intensity of the (006) and (0015) reflections, which suggests constructive interference from vertically aligned layers. The absence of peak broadening or splitting indicates that the stacking process does not introduce significant interfacial strain or disorder.^41^ These results affirm that the KOH-assisted transfer method preserves the structural integrity of Bi_2_Se_3_ and enables dual thin-film stacking without degrading crystalline order.

To further investigate the structural alignment and stacking quality of the stacked Bi_2_Se_3_ dual films, we performed cross-sectional transmission electron microscopy (TEM) imaging and nanobeam diffraction (NBD) analysis. As shown in Figure 2d, the two Bi_2_Se_3_ films are clearly distinguishable, with a well-defined interface and minimal interfacial voids, indicating successful vertical stacking without significant delamination. The top and bottom thin films remain continuous and uniform, demonstrating the effectiveness of the KOH-assisted transfer process in preserving film integrity.

Nanobeam diffraction patterns acquired from the top and bottom films (Figure 2e,f, respectively) exhibit nearly identical spot arrays, confirming the retention of crystalline order in both films post-transfer. The overlapping diffraction spots (Figure 2g) reveal a very small rotational misalignment angle of approximately 0.95°, suggesting near-aligned stacking with minimal twist-induced disorder. This small angle likely contributes to the interfacial hybridization of surface states, as discussed latter in the transport and magnetoresistance sections. These structural characterizations affirm that the mechanical stacking process produces dual thin films with high crystallinity and clean interfaces, which are critical for the stacking-induced transport phenomena in topological insulator systems.

To directly correlate the stacking-induced structural disorder with the electronic landscape, we performed room-temperature scanning tunneling microscopy (STM) and scanning tunneling spectroscopy (STS) measurements. Figure 3b presents the large-scale STM topographies, revealing a distinct morphological difference between the regions. The single thin-film Bi_2_Se_3_ exhibits characteristic terrace steps with relatively smooth surfaces, indicative of high crystalline quality. In contrast, the dual thin-film region displays a more disordered and rugged morphology. This increased surface roughness likely stems from the mechanical transfer process and the strain relaxation between the nonepitaxial layers, which introduces additional scattering centers.

**Figure 3:**
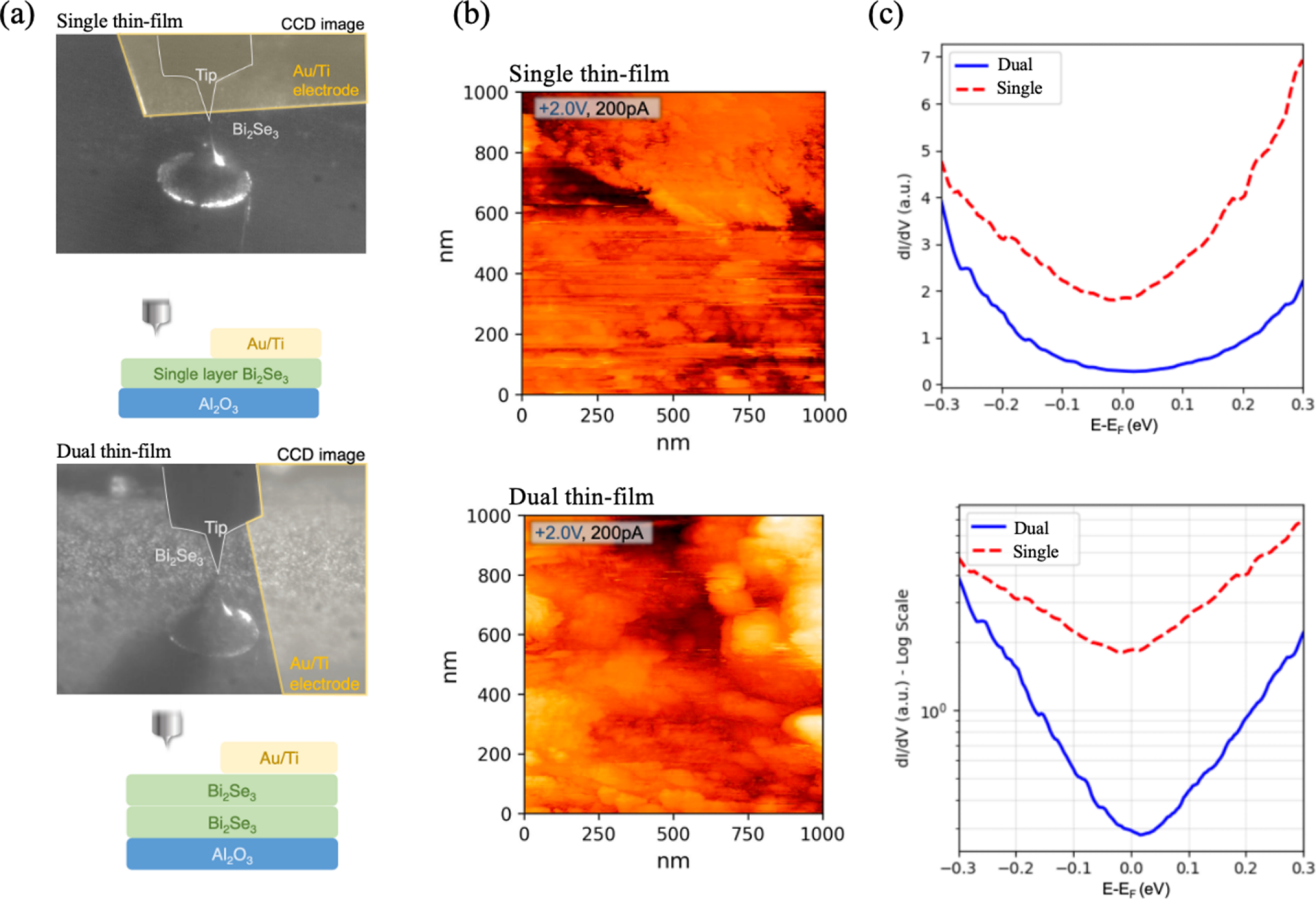
(a) Schematic illustrations and optical micrographs of the single and dual thin-film Bi_2_Se_3_ devices with Au/Ti electrodes. (b) Large-scale STM topographic images comparing the surface morphology of the single thin-film (top) and dual thin-film (bottom) regions. The dual thin-film surface shows increased roughness compared to the clean terrace steps of the single thin-film. (c) Comparison of Scanning Tunneling Spectroscopy (STS) dI/dV curves acquired from single (red dashed) and dual (blue solid) thin-film. The linear-scale plot (top) reveals the typical V-shaped Dirac cone signature in the single thin-film. The logarithmic-scale plot (bottom) highlights the significant suppression of the density of states near the Fermi level in the dual thin-film, indicative of stacking-induced disorder and carrier localization.

Probing the local electronic structure, the STS results in Figure 3c provide spectroscopic evidence of the stacking effects. The spectrum of the single thin-film (red dashed line), plotted on a linear scale (Figure 3c, top), displays a characteristic V-shaped minimum near the Fermi level (*E*_F_). This feature is the hallmark of the linear density of states associated with the pristine topological Dirac cone, confirming the preservation of coherent surface states in the single thin-film. Conversely, the dual thin-film spectrum (blue solid line) exhibits a deviation from this linear dispersion. As highlighted in the logarithmic-scale plot (Figure 3c, bottom), there is a pronounced suppression of the local density of states (LDOS) near *E*_F_ for the dual thin-film compared to the single film. This depletion of low-energy states suggests that the interfacial disorder effectively perturbs the Dirac cone, creating localized states or opening a minigap. This microscopic observation of disorder-induced DOS suppression serves as a precursor to the variable-range hopping (VRH) conduction observed in the low-temperature transport measurements.

After confirming the structural fidelity of both the single and stacked dual thin-film configurations, we examined their electronic transport properties through temperature-dependent resistance (R-T) measurements. As shown in Figure 4a, the single thin-film device maintains a low and nearly constant resistance below approximately 130 K, consistent with surface-dominated transport in topological insulators,^42^ where bulk carriers are largely frozen out at low temperatures. However, a pronounced increase in resistance emerges above ∼130 K, indicating a transition from coherent surface conduction to thermally activated bulk transport. Although more carriers are excited into the conduction band at elevated temperatures, their contribution to overall conductivity remains limited due to low mobility. This inefficiency likely arises from enhanced phonon scattering, impurity trapping, and interfacial potential fluctuations that hinder long-range carrier transport. As a result, the overall conductivity is suppressed despite increased carrier concentration, leading to a counterintuitive rise in resistance. This crossover at 130 K highlights the breakdown of surface-state coherence and the onset of a bulk-dominated conduction regime, emphasizing the critical role of structural quality and interface-induced disorder in modulating the transport behavior of topological thin films.

**Figure 4:**
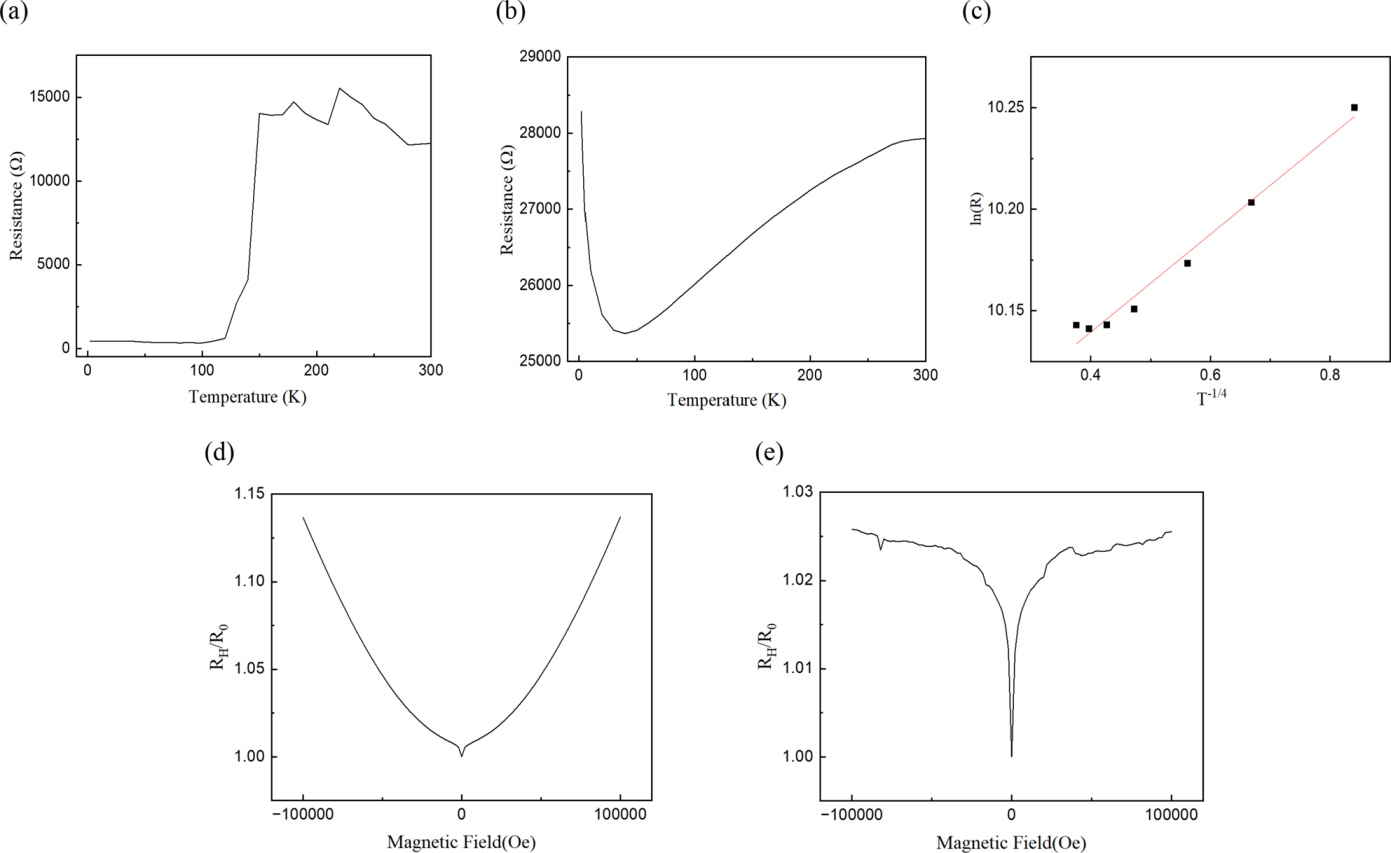
Temperature-dependent resistance (R–T) characteristics of (a) single thin-film and (b) stacked Bi_2_Se_3_ thin-film devices. (c) VRH fitting of the temperature-dependent resistance (R-T) data for the stacked Bi_2_Se_3_ thin-film device at temperatures below 50 K. The excellent fit (*R* ^2^ = 0.98) to the VRH model suggests that the conduction mechanism is dominated by hopping between localized states at low temperatures. Magnetoresistance (MR) measurements of (d) single thin-film and (e) stacked Bi_2_Se_3_ dual thin-film devices under out-of-plane magnetic fields at 2 K. The single-film sample shows a nearly linear increase in resistance, consistent with high-mobility, topologically protected surface states. In contrast, the stacked thin-film device exhibits a weak antilocalization (WAL) feature near zero field and a suppressed, nonlinear MR response at higher fields, suggesting enhanced localization and disrupted coherent surface transport due to stacking-induced disorder.

In contrast, the dual thin-film device (Figure 4b) shows a resistance minimum at the cryogenic temperature of 50 K. With the increase of temperature above 50 K, the resistance increases monotonically up to 300 K. The resistance minimum suggests a transition from coherent surface-state transport to bulk-dominated conduction as the temperature rises. Compared with the single thin film, the film-stacked sample displays a higher resistance value and a gradual increase with temperature, indicating partial suppression of surface conduction. This suppression may arise from interfacial effects introduced during stacking, such as rotational misalignment, interlayer roughness, or partial hybridization of Dirac surface states.

In Figure 4b, we observe a sharp increase in resistance below 50 K in the stacked dual thin-film device, indicating a crossover to a different conduction mechanism at cryogenic temperatures. This behavior suggests the emergence of an interfacial transport regime dominated by disorder-induced carrier localization, likely caused by stacking-induced structural inhomogeneity. In such disordered structures, long-range coherent band transport is suppressed, and charge carriers instead rely on phonon-assisted hopping between spatially and energetically localized states. This conduction behavior is well described by Mott’s three-dimensional variable-range hopping (3D VRH) model,^27–29^ which predicts a temperature-dependent resistance given by

$$R(T)=R_{0}\exp{\left(\frac{T_{0}}{T}\right)}^{1/4}$$ 1

Here, *R*_0_ is related to the intrinsic resistance scale of the system, and *T*_0_ is a characteristic temperature that depends inversely on the density of states near the Fermi level and the localization length. The fitting results for the resistance below 50 K are shown in Figure 3c. The stack dual thin-film data below 50 K exhibit strong linearity in the ln­(*R*) versus *T* ^–1/4^ plot very well, with a coefficient of determination *R* ^2^ ≈ 0.98. This fitting supports the predominance of phonon-assisted hopping between localized states in this regime, rather than transport through extended Bloch states.^43^

The comparison between single and stack dual thin-film devices (Figure 4a–c) highlights that while the single thin-film Bi_2_Se_3_ maintains coherent, topologically protected surface-state conduction at low temperatures, vertical stacking in the dual films induces a transition to a disordered regime dominated by 3D VRH transport. These findings demonstrate that stacking modifies the dimensionality of transport and introduces interlayer hybridization^23,26^ and structural disorder.^44^

To further probe the coherence and mobility of charge carriers in single and stack dual Bi_2_Se_3_ films, magnetoresistance (MR) measurements were conducted at 2 K under out-of-plane magnetic fields up to ±100 kOe. As shown in Figure 4d, the single-film device exhibits a nearly linear MR response with a maximum normalized resistance *R*_H_/*R*_0_ ≈ 1.15. This linear magnetoresistance (LMR)^2,45^ is commonly attributed to high-mobility Dirac surface states with spin-momentum locking, where conventional Lorentz scattering is suppressed and quantum coherence is preserved over long distances.

In contrast, the dual thin-film sample (Figure 4e) shows a markedly different MR response. The RH/R0 only reaches *R*_H_/*R*_0_ ≈ 1.03, and the MR profile deviates from linearity. Instead, a pronounced weak antilocalization (WAL)^46^ cusp emerges near zero field, followed by a flattened response at higher magnetic fields.

This WAL feature indicates enhanced quantum interference, but the reduced MR amplitude and loss of linear behavior imply suppression of coherent surface transport in the dual thin-film configuration. The absence of high-field linearity suggests that carrier motion is dominated by localized states rather than delocalized surface channels.^47^

These findings are consistent with the low-temperature resistance behavior discussed earlier. The emergence of 3D VRH conduction in the dual-film stacks correlates with the disruption of coherent topological channels by interfacial disorder and hybridization. Specifically, rotational misalignment and stacking-induced strain may break in-plane symmetry, leading to suppressed direct hybridization. However, they enable interlayer coupling between Dirac cones and lead to partial gapping and localization. Film transfer-related roughness and stress can also introduce random potential fluctuations, further degrading carrier coherence. The MR results reinforce the picture that nonepitaxial vertical stacking fundamentally alters the transport landscape of Bi_2_Se_3_, transforming the system from a coherent Dirac conductor into a disorder-driven hopping regime.

Across the measured temperature range, the physical origin of interlayer carrier localization is schematically illustrated in Figure 5. In vertically stacked Bi_2_Se_3_, rotational misalignment between the thin films disrupts in-plane symmetry, promoting hybridization of surface Dirac states.^48^ This hybridization opens a minigap and generates localized states near the Fermi level, suppressing topological protection and hindering coherent surface conduction. Furthermore, interfacial strain and roughness, introduced during mechanical transfer, induce spatially varying potential fluctuations that exacerbate carrier localization. At cryogenic temperatures, this disordered potential landscape inhibits long-range band conduction, forcing electrons to rely on phonon-assisted hopping between spatially and energetically localized states. The phonons provide the necessary energy to compensate for the mismatch between localized sites, enabling inelastic transitions. As described by the Mott 3D VRH model, the hopping probability decreases exponentially with decreasing temperature, consistent with the pronounced upturn in resistance observed below 50 K in our experimental results.

**Figure 5:**
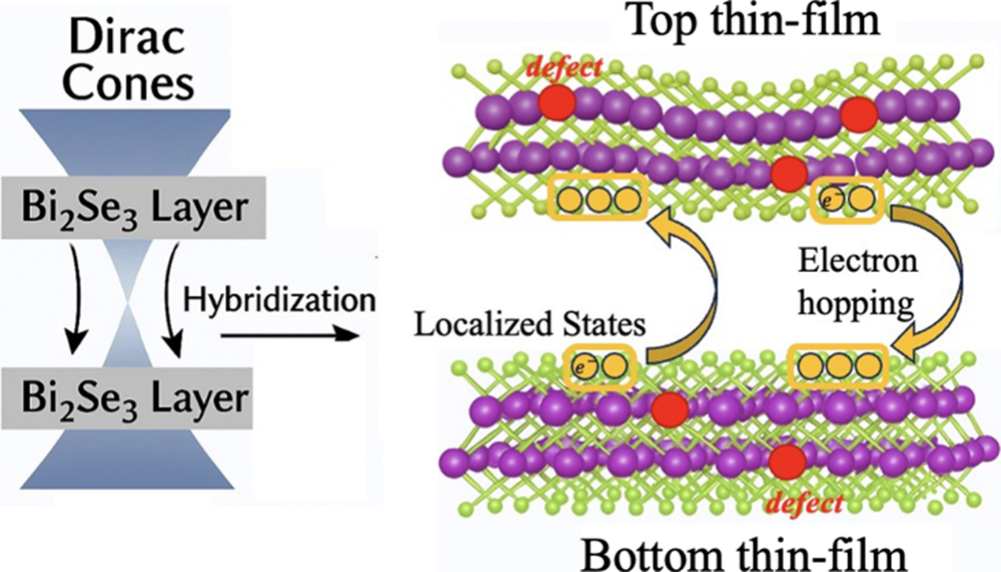
Schematic illustration of stacking-induced hybridization and electron hopping in dual thin-film Bi_2_Se_3_. Left: In vertically stacked Bi_2_Se_3_ films, hybridization of surface Dirac cones occurs due to interlayer coupling, forming localized states near the Fermi level. Right: These localized states act as trap centers that inhibit coherent surface transport. At low temperatures, electrons rely on phonon-assisted hopping between spatially and energetically localized states, resulting in variable-range hopping (VRH) conduction.

While the transfer method employed here facilitates the creation of dual-film stacks without the constraints of epitaxial growth, it lacks the precise angular control characteristic of 2D material assembly. The 0.95° misalignment observed in our samples likely arises from manual handling during the transfer process. Consequently, the resulting transport behavior is viewed as a result of stochastic interfacial disorder and hybridization rather than a precisely tuned moiré state. Future developments in automated transfer stages will be necessary to achieve reproducible angular control in 3D topological insulator films.

## 4 Conclusions

In summary, we have demonstrated a KOH-assisted mechanical transfer of CVD-grown Bi_2_Se_3_ thin film to form nonepitaxial dual thin-film Bi_2_Se_3_ stacks. Comprehensive structural, electrical, and magnetotransport characterization reveals that vertical stacking significantly alters the charge transport behavior. Single Bi_2_Se_3_ thin film preserves coherent, topologically protected surface conduction. In contrast, the dual thin-film configuration transitions to three-dimensional variable-range hopping below 50 K, accompanied by suppressed linear magnetoresistance and enhanced weak antilocalization signatures. These results indicate that interfacial disorder, rotational misalignment, and Dirac cone hybridization collectively induce carrier localization, transforming the transport regime from delocalized to hopping-type. This is the first report of systematic R-T and MR measurements on CVD-based vertically stacked Bi_2_Se_3_ dual thin films. Our findings highlight the critical role of stacking geometry in changing the electronic dimensionality of topological materials and offer a practical route toward engineered quantum transport via van der Waals heterostructure design.

## Supplementary Material



## Data Availability

All data supporting the findings of this study are available within the article and the Supporting Information.

## Notes

Section S1: KOH-assisted delamination and dual thin-film assembly; Section S2: pattern definition for van der Pauw four-probe configuration; and Section S3: VRH fitting (PDF)
