# Supplementary material for: Transition from Surface to Hopping Conduction in Stacked Nonepitaxial Bi2Se3 Dual Thin Films
Source: ACS Omega. 2026 Mar 11;11(11):17464–71. doi: 10.1021/acsomega.5c10654 (PMC13019221; doi:10.1021/acsomega.5c10654)
Supplement: Supplementary file 1 [file ao5c10654_si_001.pdf]

Supplementary Information:

Transition from Surface to Hopping Conduction in Stacked Non-Epitaxial Bi_2Se_3 Dual Thin Films

Kuan-Han Wu, [a] *Cheng-Yi Cheng,* [a] *Bo-Chien Liao,* [a] *and Jian-Jang Huang* [a,b,*]

[a] *Graduate Institute of Photonics and Optoelectronics, National Taiwan University, Taipei 10617, Taiwan*

[b] *Department of Electrical Engineering, National Taiwan University, No. 1, Roosevelt Road, Sec. 4, Taipei 106, Taiwan*

**Author e-mail address: jjhuang@ntu.edu.tw*

S1. KOH-assisted delamination and dual thin-film assembly

To fabricate dual thin-film structures, the as-grown Bi_2Se_3 thin films were transferred using a PMMA-assisted KOH delamination process. A layer of PMMA was first spin-coated onto the film surface and dried, after which the samples were immersed in diluted aqueous KOH solutions with concentrations of 13.05, 17.80, and 21.75%wt.% (Figure%S1b–d) to initiate lateral underetching.

Lower KOH concentrations (Figure%S1b) yielded larger delaminated areas but introduced clusters and wrinkles, likely caused by the prolonged etching time required for complete lift-off. At intermediate concentrations (Figure%S1c), the transfer area decreased, and surface uniformity improved slightly. The highest concentration of 21.75%wt.% (Figure%S1d) provided the best trade-off between transfer area and surface uniformity. After delamination, the PMMA-supported films were floated on the water surface and stacked onto another as-grown Bi_2Se_3 thin film. A video showing the PMMA-assisted KOH delamination and thin-film stacking process is provided as Movie%S1. The dual thin-film samples were then dried, annealed at 90%˚C for 30%min, left to stabilize at room temperature for 24%h, and finally the PMMA support layer was removed using acetone.

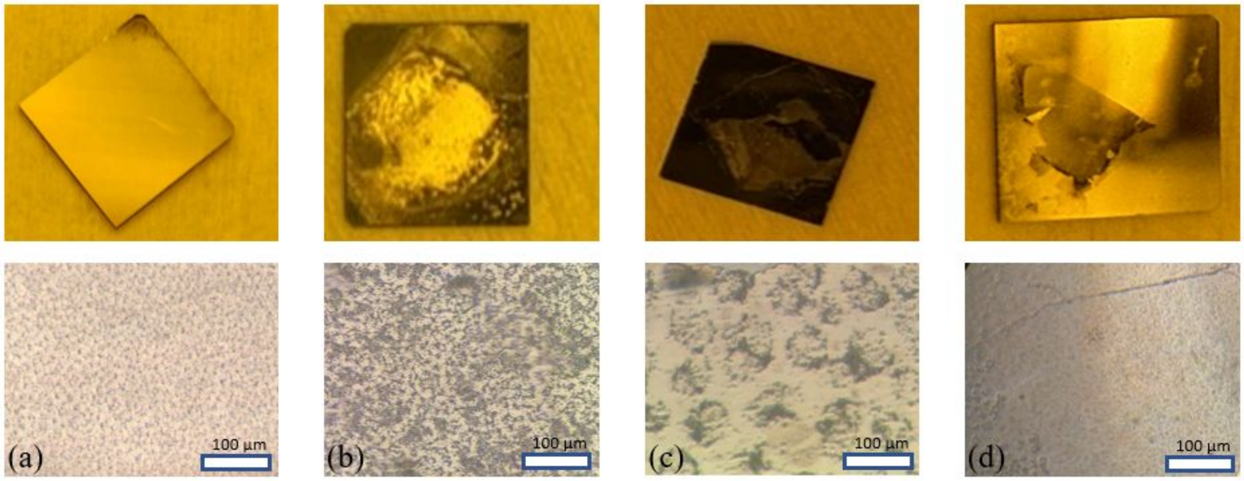

Figure S1. Optical microscopy (OM) images showing the effect of different KOH concentrations on Bi_2Se_3 film delamination and transfer quality. (a) Optical microscopic image of the as-grown Bi_2Se_3 thin film on an Al_2O_3 (0001) substrate, showing uniform morphology and full substrate coverage. Transferred thin films delaminated under (b) 13.05 wt.% KOH; (c) 17.80 wt.% KOH;

(d) 21.75 wt.% KOH. Top panels show the transferred area under visible light; bottom panels provide high-magnification OM views of the film surface. Lower concentrations yielded larger delaminated areas, while higher concentrations led to improved surface uniformity. Surface clusters and wrinkles observed at lower concentrations may be related to prolonged etching or stress relaxation during lift-off.

S2. Pattern definition for van der Pauw four-probe configuration

Bi_2Se_3 thin-film devices for electrical characterization were fabricated using standard photolithography and electron-beam evaporation techniques (Figure%S2). Ti/Au electrodes with thicknesses of 10%nm and 200%nm, respectively, were deposited by e-beam evaporation at a base pressure below 5%×%10^{-3}%Torr, followed by lift-off in acetone.

For single-layer devices, electrodes were patterned at the corners of the Bi_2Se_3 thin films to form a standard van der Pauw four-probe configuration, enabling precise resistance and Hall-effect measurements.

For dual thin-film devices, a second Bi_2Se_3 film was stacked onto the first layer using the KOH-assisted transfer method described in S1, and the electrodes were carefully aligned so that the entire contact region covered the stacked area. This alignment ensured that the measured transport properties were dominated by the bilayer region and minimized parasitic contributions from unstacked monolayer areas.

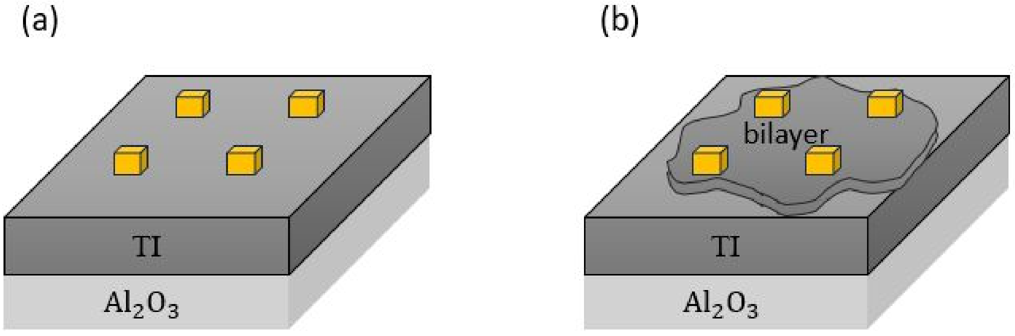

Figure S2. Bi_2Se_3 thin-film device configurations for electrical characterization. (a) Single thin-film device: Ti/Au electrodes (10 nm/200 nm) are patterned at the film corners in a standard four-probe configuration for resistance and Hall measurements.

(b) Dual thin-film device: A second Bi_2Se_3 film is stacked atop the first via a KOH-assisted transfer

process. Electrodes are precisely aligned over the dual thin-film region to enable localized investigation of vertical charge transport and interlayer coupling phenomena.

S3. VRH fitting

To further validate the transport mechanism of the dual thin-film Bi_2Se_3 device at low temperatures, the temperature-dependent resistance (R–T) data below 50%K were analyzed using the three-dimensional variable-range hopping (3D VRH) model:

$$R(T)=R_0\exp[(T_0/T)^{1/4}].$$

As shown in Figure%S3, a linear relationship was obtained when plotting ln(R) versus $T^{-1/4}$, consistent with the 3D VRH conduction mechanism. The fitting was performed using Origin, yielding a high coefficient of determination (R^2=0.98) and reliable fitting parameters (see inset table). These results confirm that the upturn in resistance observed below 50%K originates from carrier localization and hopping conduction.

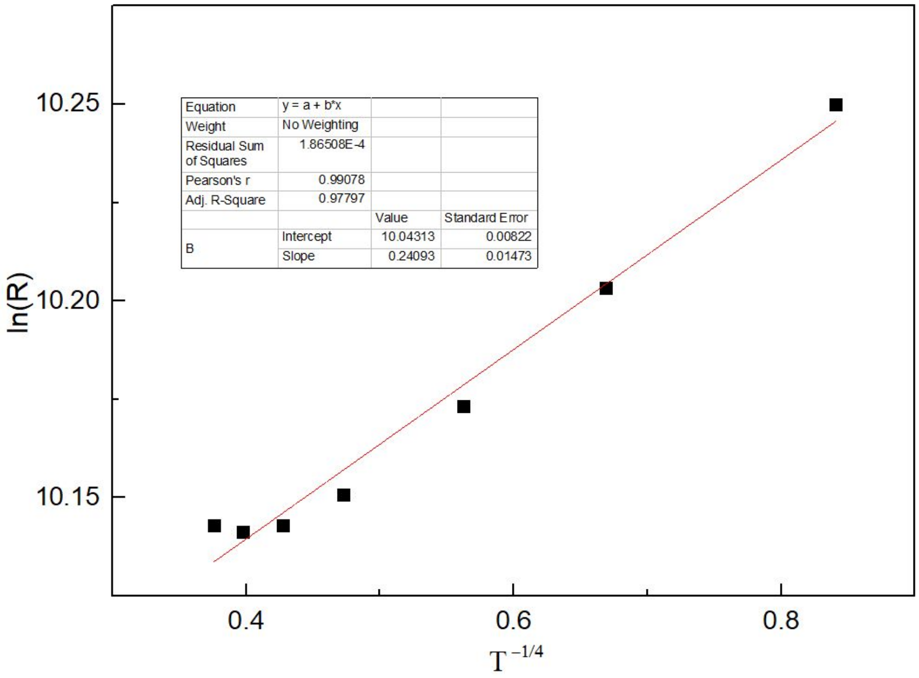

Figure S3. Three-dimensional variable-range hopping (3D VRH) fitting of the dual thin-film Bi_2Se_3 device below 50%K. Experimental data are plotted as ln(R) versus $T^{-1/4}$, and the red line represents the VRH fit according to $R(T)=R_0\exp[(T_0/T)^{1/4}]$. The high coefficient of determination

(R^2=0.98) and fitting parameters (inset table) confirm the validity of the VRH conduction mechanism.
